# Sustained remission of psychotic symptoms secondary to hypothyroidism (myxedema psychosis) after 6 months of treatment primarily with levothyroxine: a case report

**DOI:** 10.1186/s13256-022-03626-x

**Published:** 2022-10-20

**Authors:** Eric C. Chan

**Affiliations:** 1 Department of Psychiatry, University of Calgary, 1403 - 29th Street NW, Calgary, AB T2N 2T9 Canada; 2 Mathison Centre for Mental Health, Calgary, Canada

**Keywords:** Myxedema madness, Myxedema psychosis, Hypothyroidism, Psychotic disorders, Thyroxine, Antipsychotic agents, Thyroid-stimulating hormone

## Abstract

**Background:**

Psychotic symptoms associated with hypothyroidism, also known as “myxedema psychosis,” are a treatable cause of psychosis often associated with complete recovery. While most cases receive both thyroxine and a short course of antipsychotics, some reports indicate that symptoms can resolve without antipsychotic treatment, though follow-up in these cases has often been short or not reported. This is one of the first case reports demonstrating sustained remission of psychotic symptoms at 6 months in a case of myxedema psychosis treated with minimal antipsychotic medication.

**Case presentation:**

We describe the case of a 40-year-old Caucasian woman who was brought to hospital with a 7-day history of anxiety and decreased sleep and 1 day of disorganized speech, paranoid delusions, and auditory hallucinations. After being admitted to psychiatry for management, screening blood work revealed elevated thyroid-stimulating hormone. The patient was initiated on treatment with levothyroxine and low doses of antipsychotics. Her symptoms resolved on the third day of her admission with ongoing symptomatic remission at 6 months follow-up.

**Conclusions:**

The identification of myxedema psychosis is important owing to the implications on treatment and prognosis of the disorder. Our case suggests that sustained symptom resolution may occur with little to no antipsychotic treatment, though these findings are preliminary and additional study is needed before definitive conclusions on the optimal approach can be made.

## Background

Psychotic symptoms associated with hypothyroidism, referred to in previous literature and in this manuscript as “myxedema psychosis,” have been described in many case reports in the medical literature. One of the earliest publications on the subject was prepared by Asher, who described 14 cases in 1949 [1]. Since then, many other cases of myxedema psychosis have been described. A recent systematic review identified 71 case reports on myxedema psychosis published between 1980 and 2019 [2]. Complete recovery was observed in more than 90% of cases, with the majority of cases receiving oral thyroxine and short-term antipsychotics. In some instances, myxedema psychosis was treated with thyroid replacement without antipsychotics [3–7]. Complete recovery was observed in these cases, though the follow-up time in each case was brief or unspecified. We describe a case of myxedema psychosis in a 40-year-old Caucasian woman who presented with a 7-day history of anxiety and decreased sleep and a 1-day history of delusions and disorganized speech. She was identified as having hypothyroidism at the time of admission and was treated with levothyroxine and a small number of low doses of antipsychotics. Her symptoms resolved the following day with no recurrence of symptoms after 6 months.

## Case presentation

A 40-year-old Caucasian woman with a history of attention deficit hyperactivity disorder (ADHD) and depression was brought to hospital with a 7-day history of anxiety and little to no sleep. In the day prior to presentation, she exhibited disorganized speech and delusions that her mother was going to die. In the emergency department, she was overinclusive and talking nonsensically. She was paranoid of security staff, endorsed auditory hallucinations, believed she was at a “séance,” and appeared to be responding to internal stimuli. Consequently, she was admitted to psychiatry for further workup and management. Of note, an emergency physician noted organized speech when she was initially seen, though this was not observed by the psychiatry team.

Her thyroid-stimulating hormone (TSH) at the time of admission was 123.90 mIU/L (reference range 0.20–4.00 mIU/L). Complete blood count, electrolytes, blood glucose, and creatinine were all normal. Coronavirus disease 2019 (COVID-19) swab was negative. The patient was on no medications prior to admission and had no history of hypothyroidism or other medical conditions. She had previously been prescribed stimulant medications for ADHD but had not taken any stimulants for months owing to previous intolerance. She denied any substance use, including alcohol and nicotine, leading to her presentation, and urine toxicology screen was negative for amphetamines or other substances.

The patient was living alone in an apartment prior to being brought to hospital. She had a boyfriend and no children. She was unemployed at the time of admission but was to start a new job working with immigrants. Her mother had a history of hypothyroidism, and the patient was not sure if she had any other family history of thyroid or autoimmune disorders. No family history of other conditions was identified.

The patient’s symptoms fluctuated when she was seen the day following admission. She initially was more organized and was able to describe experiencing persecutory and religious delusions in the week prior to admission. Nonetheless, she continued to experience delusional beliefs that her mother was going to die. Later in the day, she was noted to be more tangential and was unable to answer questions.

When seen by the endocrinology consult team, the patient reported experiencing cold intolerance, fatigue, dry skin, poor appetite, and hair loss. She did not notice any weight gain. She reported feeling her voice was weaker and that she felt like she had a frog in her throat. The patient reported that she had previously been diagnosed with Hashimoto’s thyroiditis but that this had not been felt to be sufficiently severe to require treatment at that time. While documentation from a previous assessment is unavailable, the patient was noted to have an elevated anti-thyroid peroxidase result (67.1 kIU/L, reference range 0.0–34.0 kIU/L) approximately 5 years prior, with TSH within normal limits, and this may have been why she had been evaluated as not requiring treatment. On physical examination, she had a slightly enlarged thyroid gland, hyperreflexia, and mild tremor. Notably, delayed relaxation or diminished deep tendon reflexes are typically observed in previous case reports of myxedema psychosis [2], and it is unclear why hyperreflexia and mild tremor were observed on physical examination in this case. It is possible that this finding was erroneous; however, further description of findings in cases of myxedema psychosis may provide more clarity. Cardiac examination was unremarkable, and no nail changes were observed. Additional blood work showed decreased total triiodothyronine (T3) of 0.9 nmol/L (reference range 1.1–2.8 nmol/L), free T3 of 2.8 pmol/L (reference range 3.5–6.5 pmol/L), and free thyroxine (T4) of 4.6 pmol/L (reference range 10.0–25.0 pmol/L). Thyroid peroxidase (TPO) antibodies were elevated at 147.6 kIU/L (reference range 0.0–34.0 kIU/L). Anti-thyroglobulin antibody and thyroglobulin levels were within normal limits. Neck ultrasound described a heterogeneous, hyperemic, non-enlarged thyroid gland with hypoechoic nodules abutting the posterior aspect and inferior aspects of the thyroid gland. On the basis of the presence of abnormal thyroid function in the context of psychosis, a diagnosis of myxedema psychosis was made.

The patient received one dose of haloperidol 5 mg and lorazepam 2 mg at the time of admission. She received quetiapine 50 mg and lorazepam 2 mg on the first evening of her admission and quetiapine 25 mg on the third evening of her admission. Levothyroxine 100 μg daily was initiated on the 2nd day of her admission, and this was increased to 200 μg daily after 2 days. The only other medication she received while in hospital was zopiclone 7.5 mg the evening of the 4th day of her admission.

The day after initiation of levothyroxine (the 3rd day of the patient’s admission), the patient was organized with no evidence of psychosis. The patient showed no further evidence of psychosis during admission and was discharged on the 5th day of her admission. Her TSH had decreased to 73.23 mIU/L (reference range 0.20–4.00 mIU/L) the day prior to discharge, and on the advice of the endocrinology service, her levothyroxine was decreased to 100 μg daily at the time of discharge.

At follow-up 6 months post-hospitalization, she has not required any psychotropic medications and has remained stable and asymptomatic in the community.

## Discussion

In addition to exhibiting many features observed in previous descriptions of myxedema psychosis, this case is important as it demonstrates that rapid resolution of symptoms and sustained symptomatic remission can occur when myxedema psychosis is treated with minimal antipsychotic medication. Consistent with previous cases, we observed complete resolution of symptoms with thyroid hormone replacement. While the patient received antipsychotic medication on three occasions during admission, we note that only the dose of haloperidol 5 mg is a typical antipsychotic dose used for the treatment of acute psychosis. While quetiapine 25 and 50 mg may be used as an initial dose, the typical dose range of quetiapine used for psychosis is between 400 and 800 mg/day [8]. Given the patient’s rapid resolution and sustained remission of symptoms, this case suggests that antipsychotic medication may not be necessary in cases of myxedema psychosis, if the condition is identified and treated with thyroid hormone replacement early. This finding highlights the need for further investigation to determine the role of antipsychotic medication in the management of myxedema psychosis, especially since 92% of previous case reports of myxedema psychosis involved treatment with antipsychotics, many for several weeks or longer [1].

As has been described in many previous case reports of myxedema psychosis, our patient experienced complete resolution of symptoms. In this instance, the patient presented with complaints of psychiatric symptoms, with signs and symptoms of hypothyroidism noted only after abnormal blood work prompted a referral to endocrinology. This observation has been observed in previous case reports, with 26% of recent case reports noting the absence of hypothyroidism signs and 37% noting the absence of hypothyroid symptoms [2]. Consequently, this report adds to the existing literature that suggests that screening of thyroid function may be an important component of initial workup for first presentations of psychosis. As existing data are limited primarily to case reports, further study is needed to improve our understanding of the incidence and outcomes of this disease.

The possibility that symptoms of psychosis due to hypothyroidism psychosis can resolve completely with little or no antipsychotic medication has important implications in the clinical management of this condition. As with many other medications, antipsychotics are associated with a range of potential side effects, including sedation, extrapyramidal symptoms, anticholinergic effects, sexual side effects, and metabolic side effects, among others [9]. If psychotic symptoms occurring in the context of myxedema psychosis can resolve without antipsychotics, it is possible that antipsychotic treatment, and the associated risks of side effects, can be avoided without negatively impacting the patient’s long-term outcomes. Systematic studies evaluating different treatment approaches to myxedema psychosis are necessary, however, before definitive recommendations can be made on the management of this disorder.

## Conclusions

Myxedema psychosis represents a potentially reversible cause of psychotic symptoms with good response to thyroid replacement therapy. We describe a case of myxedema psychosis in which the patient experienced full resolution of psychotic symptoms with thyroid hormone replacement and minimal treatment with antipsychotic medication. As the patient remains stable and asymptomatic 6 months post-discharge, this case suggests that antipsychotic medication may not always be necessary in the treatment of myxedema psychosis. Additional study is necessary, however, before this suggestion could be considered routine clinical practice.

## Data Availability

Not applicable.
